# Improving Outcomes of Chemotherapy: Established and Novel Options for Myeloprotection in the COVID-19 Era

**DOI:** 10.3389/fonc.2021.697908

**Published:** 2021-07-08

**Authors:** Gary H. Lyman, Nicole M. Kuderer, Matti Aapro

**Affiliations:** ^1^ Public Health Sciences and Clinical Research, Fred Hutchinson Cancer Research Center, Seattle, WA, United States; ^2^ Department of Medicine, University of Washington, Seattle, WA, United States; ^3^ Advanced Cancer Research Group, Seattle, WA, United States; ^4^ Genolier Cancer Center, Clinique de Genolier, Genolier, Switzerland

**Keywords:** anemia, chemotherapy, COVID-19, myeloprotection, myelosuppression, neutropenia, thrombocytopenia

## Abstract

Chemotherapy-induced damage of hematopoietic stem and progenitor cells (HPSCs) often results in myelosuppression that adversely affects patient health and quality of life. Currently, chemotherapy-induced myelosuppression is managed with chemotherapy dose delays/reductions and lineage-specific supportive care interventions, such as hematopoietic growth factors and blood transfusions. However, the COVID-19 pandemic has created additional challenges for the optimal management of myelosuppression. In this review, we discuss the impact of this side effect on patients treated with myelosuppressive chemotherapy, with a focus on the prevention of myelosuppression in the COVID-19 era. During the COVID-19 pandemic, short-term recommendations on the use of supportive care interventions have been issued with the aim of minimizing the risk of infection, reducing the need for hospitalization, and preserving limited blood supplies. Recently, trilaciclib, an intravenous cyclin-dependent kinase 4 and 6 inhibitor, was approved to decrease the incidence of myelosuppression in adult patients when administered prior to platinum/etoposide-containing or topotecan-containing chemotherapy for extensive-stage small cell lung cancer (ES-SCLC). Approval was based on data from three phase 2 placebo-controlled clinical studies in patients with ES-SCLC, showing that administering trilaciclib prior to chemotherapy significantly reduced multilineage myelosuppression, with patients receiving trilaciclib having fewer chemotherapy dose delays/reductions and myelosuppression/sepsis-related hospitalizations, and less need for supportive care interventions, compared with patients receiving placebo. Several other novel agents are currently in clinical development for the prevention or treatment of multilineage or single-lineage myelosuppression in patients with various tumor types. The availability of treatments that could enable patients to maintain standard-of-care chemotherapy regimens without the need for additional interventions would be valuable to physicians, patients, and health systems.

## Introduction

Despite significant progress in precision medicine and immunotherapy, chemotherapy remains the cornerstone of treatment for most cancers. However, chemotherapy is often associated with severe side effects that can affect the patient’s quality of life (QoL), often compromising the patient’s willingness to continue with, and successfully complete, treatment (1, 2).

Although cytotoxic chemotherapy drugs affect all dividing cells, tumor cells typically proliferate faster than non-tumor cells, rendering tumor cells particularly sensitive to the antineoplastic effects of chemotherapy. However, because cytotoxic chemotherapies are not selective, serious toxicity to healthy organs also occurs. Proliferating hematopoietic stem and progenitor cells (HSPCs) in the bone marrow are particularly susceptible to chemotherapy-induced damage. Consequently, one of the most common side effects of chemotherapy is myelosuppression, which typically manifests as neutropenia, anemia, thrombocytopenia, and/or lymphopenia. The magnitude of damage to neutrophils, red blood cells (RBCs), platelets, and/or lymphocytes depends on the chemotherapy regimen used and baseline patient characteristics (1, 3–5).

Chemotherapy-induced damage of HSPCs leads to acute and long-term negative effects, placing patients at significant risk of serious infections, and even death (6, 7). The immunosuppressed state that results from cancer and anticancer treatment may also put patients at an increased risk of contracting infections such as COVID-19 and developing serious complications (8–10).

Typically, chemotherapy-induced myelosuppression is managed with dose delays or reductions, which reduce chemotherapy dose intensity and potentially limit therapeutic efficacy (11, 12), and with supportive intervention with hematopoietic growth factors (granulocyte colony-stimulating factors [G-CSFs] and erythropoiesis-stimulating agents [ESAs]) and blood transfusions. However, these interventions are lineage specific, are usually used after adverse events (AEs) have occurred and introduce their own set of side effects.

## Current Supportive Care Measures for Myelosuppression

Myelosuppression represents a considerable economic and humanistic burden, incurring substantial financial costs (13) while negatively affecting patients’ QoL owing to symptoms such as fatigue and concerns over infection (2).

Administration of G-CSFs such as filgrastim, lenograstim, or pegfilgrastim (and approved biosimilars) is the mainstay of prophylactic treatment to reduce the risk of chemotherapy-induced neutropenia (CIN) (14, 15). In general, primary G-CSF prophylaxis (during cycle 1 of chemotherapy) is recommended for regimens carrying a ≥ 20% risk of febrile neutropenia (FN). For intermediate- or low-risk regimens, primary prophylaxis with G-CSF is not typically recommended, with G-CSF typically reserved for subsequent chemotherapy cycles after the onset of FN; however, a patient’s age and coexisting morbidities should be considered for those receiving intermediate-risk regimens (10–20% risk of FN) (14–16). Although primary G-CSF prophylaxis can reduce the incidence of FN and infection-related mortality (17), it is also associated with bone or musculoskeletal pain that often requires additional treatment with non-steroidal anti-inflammatory drugs, antihistamines, and opioids (17, 18).

Treatment of chemotherapy-induced anemia (CIA) is based on the use of ESAs with or without iron, iron treatment alone, or RBC transfusions, each of which are administered after the onset of anemia and are associated with certain risks or limitations (19). ESAs are effective in approximately 60% of patients, and inappropriate use carries a risk of thromboembolic disease; however, despite previous concerns that ESAs may increase mortality, there is no evidence indicating a detrimental effect of ESAs on clinical outcomes when used according to the label and published guidelines (19, 20). Available evidence also suggests that intravenous iron does not increase the risk of tumor progression, although its long-term safety in oncology patients is not yet fully established. Finally, RBC transfusions are associated with an increased risk of thrombotic events, occult infection, immunosuppression, alloimmunization, and transfusion reactions, in addition to being a temporary solution (19, 21).

If no other underlying cause of thrombocytopenia can be identified, the only treatments for chemotherapy-induced thrombocytopenia (CIT) are platelet transfusion and/or chemotherapy dose modification (3). Prophylactic platelet transfusions are recommended in patients without clinically significant bleeding if platelet counts are < 10,000/µL (or 10,000–20,000/µL in patients with additional risk factors) (3, 22). Platelet transfusions are associated with all types of blood transfusion reactions, most commonly acute transfusion reaction (allergic or febrile non-hemolytic reactions). Other, less common AEs include transfusion-related acute lung injury, hemolysis, and bacterial sepsis, although the latter has become rare since the introduction of bacterial screening (22).

Overall, the management of myelosuppression with the current treatment armamentarium remains suboptimal, and an unmet need remains for a treatment that can minimize side effects by providing multilineage protection from cytotoxic damage, particularly among high-risk patients.

## Managing Myelosuppression in a COVID-19 and a Post–COVID-19 Setting

In December 2019, an outbreak of acute respiratory syndrome emerged in Wuhan, China. Now known as the coronavirus disease-19 (“COVID-19”), a novel beta-coronavirus caused by severe acute respiratory syndrome coronavirus 2 (SARS-CoV-2), this disease rapidly spread across the globe (23). By the end of January 2020, the World Health Organization (WHO) had declared a global public health emergency, and COVID-19 was declared a pandemic in March 2020 (24). As of May 11, 2021, there had been over 150 million confirmed cases worldwide, with more than 3 million deaths (25). The SARS-CoV-2 virus binds to angiotensin-converting enzyme 2 receptors on epithelial cells in the respiratory tract, thus allowing cell entry, followed by replication and migration through the airways and into the lungs. Rapid replication of the virus in the lungs sometimes triggers a strong immune response, resulting in the release of various cytokines and chemokines (23, 26). The clinical presentation of COVID-19 ranges from asymptomatic to severe illness and death, with fever, cough, fatigue, and shortness of breath among the most common symptoms. Most patients start with milder symptoms and progress to moderate or severe disease over the course of a week, with some patients developing complications such as sepsis and acute respiratory distress syndrome (ARDS) (23).

Several reports from China and the US suggest that patients with cancer are more susceptible to COVID-19 infection and more likely to experience severe complications, which often coincide with severe immunosuppression (8, 9, 27, 28). However, evidence regarding whether patients undergoing anticancer treatment are more susceptible to COVID-19 than those who are not receiving active treatment is conflicting (9, 29). To explore factors affecting outcomes and effective treatment approaches for patients with cancer and COVID-19, the COVID-19 and US Cancer Consortium (CCC19) registry database was formed in March 2020 to collect de-identified patient data from US and international sites (27, 30, 31). Additional monitoring projects have been launched in other countries, including the UK Coronavirus Cancer Monitoring Project (UKCCMP) (32). Although reports from the CCC19 registry and UKCCMP found no evidence that patients receiving anticancer treatment are at an increased risk of COVID-19–related mortality (31, 32), data from China suggest that hospitalization and repeated hospital visits for cancer treatment and monitoring are potential risk factors for hospital-acquired COVID-19 infection (29). Continuing to shield patients with cancer from exposure to COVID-19 by self-isolation and by minimizing the number of visits to health care facilities is therefore warranted (32).

Issues relating to myelosuppression in patients with cancer are particularly pertinent in the context of COVID-19, as changes in hematologic parameters are common in infected patients, particularly in severe cases (33, 34). Like other coronaviruses, SARS-CoV-2 can infect bone marrow cells and inhibit hematopoiesis, leading to complications such as lymphopenia and thrombocytopenia (33, 34). Additionally, thrombocytopenia in patients with COVID-19 may result from other mechanisms, such as platelet destruction by the immune system, and/or platelet consumption due to aggregation and formation of microthrombi in the lungs (34). Although neutropenia is rarely reported because of COVID-19 infection (35), patients with COVID-19 and severe neutropenia (SN) may be more susceptible to complications and poorer outcomes. One study of 63 oncology patients admitted with COVID-19 in Spain found that SN was an independent risk factor for mortality on multivariate analysis, and neutropenia was more common among patients with COVID-19 who developed ARDS (36).

Another important consequence of the COVID-19 pandemic is its impact on blood product supplies, with anxiety regarding infection and social isolation potentially leading to a reduction in blood donations (33). Consequently, there is a need to consider additional measures to prevent and mitigate the consequences of myelosuppression and to facilitate continued administration of effective chemotherapy (37). The National Comprehensive Cancer Network (NCCN^®^) issued short-term recommendations specific to issues with COVID-19 in managing myelosuppression in patients with cancer (37). These guidelines acknowledge that some issues, such as potentially limited blood supplies, the possible impact of COVID-19 on patients with an active infection, and the need for measures that might shorten or prevent hospitalization, were not adequately addressed by existing guidance. Accordingly, in the short-term recommendations, the threshold for prophylactic G-CSF has been changed to include intermediate-risk as well as high-risk cases. Additionally, therapeutic G-CSF is recommended for all patients developing FN who have not previously received G-CSF, rather than only those at risk of developing complications. Considering the potential for blood shortages, the use of ESA therapy with or without iron supplementation should be broadened to manage anemia in patients with cancer requiring blood transfusion support. If blood supplies are severely limited, ESAs may be considered when transfusion support is not available or if patients refuse transfusion. For oncology patients with thrombocytopenia, the threshold for platelet transfusion may be lowered to a platelet count of < 10,000, with some centers using < 20,000 for outpatients, modified for patients with bleeding. Prophylactic antifibrinolytics and thrombopoietin mimetics may be considered for some patients based on concerns over CIT and bleeding in the context of a possible platelet supply shortage, and over potential risks from exposure to health care facilities for platelet transfusion (37).

The European Society for Medical Oncology (ESMO) has also issued guidance on the adaptation of supportive care strategies for patients with cancer during the COVID-19 pandemic (38). As FN can result in the need for hospitalization, ESMO recommends that physicians consider using regimens with a lower risk of FN in patients who are not being treated with curative intent. ESMO also suggests expanding the indication of G-CSF after chemotherapy to lower the risk of FN but acknowledges that this may require additional outpatient visits. Anemia should be treated primarily according to the level of symptoms rather than specific hemoglobin thresholds, and ESAs (particularly long-acting formulations) given to chemotherapy-treated patients with symptomatic anemia to reduce the number of hospital visits. Administration of RBC transfusions is the preferred option for patients with severe anemia-related symptoms and a need for immediate hemoglobin and symptom improvement (38).

Like NCCN and ESMO recommendations, the American Society of Clinical Oncology (ASCO) recognizes that, during the COVID-19 pandemic, it may be reasonable for patients at lower risk of FN to be prescribed growth factors (39). ASCO also recommends that ESAs be considered for patients with serious and/or symptomatic cancer- or treatment-related anemia when deemed safe, and that transfusions be administered in accordance with usual practice but with consideration of the availability of local blood supplies (39).

Despite the considerable need to minimize COVID-19–associated complications and hospitalizations, the side effects associated with lineage-specific interventions for myelosuppression must still be considered in both a COVID-19 and a post–COVID-19 setting. For example, both the NCCN and ESMO recommendations continue to stress caution regarding the increased risk of thrombosis with ESAs and thrombopoietin mimetics (37, 38). The potential for thrombotic adverse effects is particularly relevant as COVID-19 has been shown to cause a profoundly prothrombotic state that is recognized as a major cause of morbidity and mortality (40–42). Although the precise pathophysiology underlying the hypercoagulable state in COVID-19 is unclear, the mechanisms appear to be unique to SARS-CoV-2, and center around the interplay between inflammation and thrombosis (41). Ultimately, the prothrombotic effect of ESAs and thrombopoietin mimetics, combined with the prothrombotic milieu observed in COVID-19, could lead to additive adverse effects (41). In addition to the potential risks of ESAs, their effectiveness may be compromised in anemic patients with COVID-19, since the efficacy of ESAs is markedly impaired under states of inflammation (43, 44). Finally, there is also some evidence that G-CSF administration could lead to more severe outcomes with COVID-19, potentially due to increased pulmonary inflammation and macrophage activation (37, 45), and that patients with severe COVID-19 infection may develop a “cytokine storm” characterized by increased levels of inflammatory markers, including G-CSF (46). Considering these limitations, the availability of a treatment that can proactively protect against myelosuppression and enable patients to remain on chemotherapy without the need for growth factors or transfusions would be particularly valuable.

Given the unmet need for better treatments for myelosuppression, several agents are in clinical development for the treatment of one or more cytopenia in patients with various cancer types (**Table 1**).

**Table 1:** Agents under clinical investigation for the treatment of myelosuppression.

Drug name	Molecule type	Route of administration	MoA	Clinical findings	Ongoing trials for myelosuppression
Trilaciclib (COSELA^™^)	CDK4/6 inhibitor	IV	Transiently arrests CDK4/6-dependent HSPCs and lymphocytes during chemotherapy exposure to protect them from chemotherapy-induced damage (myeloprotection)	Significant reduction in DSN in cycle 1 and SN versus placebo in patients with SCLC Less hematologic toxicity Reduced use of supportive care interventions and myelosuppression-related hospitalizations Improved HRQoL No impact on antitumor efficacy of chemotherapy regimens used in SCLC Significant improvement in OS in patients with TNBC	I-SPY 2 neoadjuvant trial in patients with breast cancer (NCT01042379) Phase 3—prevention of myelosuppression in CRC (NCT04607668) EAP in SCLC (NCT04504513)
Plinabulin	Marine derived, small molecule	IV	Microtubule destabilizer; boosts number of HSPCs and increases the ability of dendritic cells to activate T cells	Similar alleviation of CIN versus pegfilgrastim; alleviation of CIT Less bone pain and improved QoL versus pegfilgrastim	Phase 3—prevention of CIN in advanced NSCLC (NCT02504489) Phase 3—prevention of CIN in solid tumors (NCT03102606) Phase 3—prevention of CIN in breast cancer (NCT03294577)
Avatrombopag (DOPTELET^®^)	Thrombopoietin receptor agonist	Oral	Stimulates megakaryocyte proliferation and differentiation, resulting increased platelet production	No results available yet; granted Orphan Drug Designation for the potential treatment of CIT	Phase 3—prevention of CIT in ovarian, lung, and breast cancers (NCT03471078)
Romiplostin (NPLATE^®^)	Fc–peptide fusion protein	SC	Binds to and activates thrombopoietin receptor, leading to increased platelet production	93% of patients experienced correction of platelet count within 3 weeks; most resumed chemotherapy without recurrence	Phase 2—prevention of CIT in hematologic malignancies (NCT02052882)
ALRN-6924	Cell-permeating alpha-helical peptide	IV	Dual MDMX and MDM2 inhibitor; disrupts MDMX/MDM2–p53 interaction, resulting in cell-cycle arrest of wild-type p53 cells	Preclinical and Phase 1b clinical data show mitigation of topotecan-induced anemia, neutropenia, and thrombocytopenia	Phase 1/2—prevention of CIN, CIA, and CIT in SCLC (NCT04022876)
Roxadustat	Hypoxia-inducible factor prolyl hydroxylase inhibitor	Oral	Promotes erythropoiesis by increasing endogenous erythropoietin production	No clinical results available yet	Phase 2—prevention of CIT in non-myeloid malignancies (NCT04076943)

CDK4/6, cyclin-dependent kinase 4 and 6; CIA, chemotherapy-induced anemia; CIN, chemotherapy-induced neutropenia; CIT, chemotherapy-induced thrombocytopenia; CRC, colorectal cancer; DSN, duration of severe neutropenia; EAP, expanded access program; HRQoL, health-related quality of life; HSPC, hematopoietic stem and progenitor cell; IV, intravenous; MDM2, mouse double minute 2; MDMX, mouse double minute X; MoA, mechanism of action; NSCLC, non-small cell lung cancer; OS, overall survival; QoL, quality of life; SC, subcutaneous; SCLC, small cell lung cancer; SN, severe neutropenia; TNBC, triple-negative breast cancer.

## Trilaciclib

In February 2021, trilaciclib (COSELA^™^), an intravenous cyclin-dependent kinase 4 and 6 (CDK4/6) inhibitor, was approved by the US Food and Drug Administration to decrease the incidence of chemotherapy-induced myelosuppression in adult patients when administered prior to a platinum/etoposide- or topotecan-containing regimen for extensive-stage small cell lung cancer (ES-SCLC) (47). Trilaciclib has now been integrated into the NCCN Hematopoietic Growth Factor guidelines, with category 2A/B recommendations for use as a prophylactic option to decrease the incidence of chemotherapy-induced myelosuppression when administered before (prophylactic G-CSF may be administered after cycle 1) platinum/etoposide ± immune checkpoint inhibitor-containing regimens or a topotecan-containing regimen for ES-SCLC (48). When administered prior to chemotherapy, trilaciclib arrests HSPCs and lymphocytes, which are dependent on CDK4/6 activity for proliferation, in the G1 phase of the cell cycle. Transient arrest of these cells in the presence of chemotherapy protects them from chemotherapy-induced damage (myeloprotection) and has the potential to enhance immune responses (49–53). Following chemotherapy, normal hematopoiesis can resume, with administration of trilaciclib prior to chemotherapy resulting in accelerated hematologic recovery (49, 50).

Trilaciclib differs from other approved CDK4/6 inhibitors in its route of administration, dosing schedule, short half-life, and intended use with chemotherapy (53). Palbociclib, ribociclib, and abemaciclib are administered orally, and dosed chronically to inhibit CDK4/6-dependent tumor cell proliferation. By contrast, trilaciclib is administered as a 30-minute intravenous infusion within 4 hours prior to the start of chemotherapy on each day that chemotherapy is administered, to target CDK4/6-dependent HSPCs and lymphocyte populations. Cell cycle arrest is transient due to the daily dosing and short half-life of trilaciclib (47, 51). To date, trilaciclib has primarily been studied in patients with ES-SCLC, a CDK4/6-independent tumor. SCLC cells continue to replicate even when treated with a CDK4/6 inhibitor and therefore remain susceptible to the cytotoxic effects of chemotherapy when trilaciclib is administered.

Data from three randomized phase 2 clinical trials evaluating trilaciclib in patients with ES-SCLC have shown that addition of trilaciclib prior to chemotherapy can reduce the risk of myelosuppression, as measured by multiple endpoints (54–56). The pivotal trial was designed to evaluate the effects of trilaciclib on myelosuppression in patients with newly diagnosed ES-SCLC treated with carboplatin and etoposide (E/P) in combination with atezolizumab, and to investigate if the potential immune-enhancing effects of trilaciclib (52) would translate to an improvement in the antitumor efficacy of atezolizumab (54). Administering trilaciclib before chemotherapy clinically and significantly reduced both the duration of SN (DSN) in cycle 1 and the percentage of patients with SN. DSN, which is used as a primary endpoint to assess the clinical efficacy and biosimilarity of G-CSFs, is a clinically relevant endpoint because the risk of infection is proportional to the severity and duration of neutropenia (57). Trilaciclib administered prior to E/P plus atezolizumab also resulted in lower rates of grade 3/4 anemia and thrombocytopenia, fewer supportive care requirements, and an improved overall safety profile for E/P plus atezolizumab, primarily due to a reduction in high-grade hematologic AEs attributable to chemotherapy. Additionally, the trilaciclib group had fewer all-cause dose reductions than the placebo group, suggesting that the administration of trilaciclib prior to chemotherapy helped to facilitate delivery of chemotherapy according to the standard dose and schedule. Flow cytometry data and analysis of the T-cell repertoire indicated that administering trilaciclib prior to E/P plus atezolizumab enhanced T-cell activation; however, survival outcomes were similar for patients receiving trilaciclib or placebo (54).

The myeloprotective effects of trilaciclib were also shown in an exploratory proof-of-concept study, in which patients with newly diagnosed ES-SCLC received trilaciclib or placebo administered once daily before first-line E/P (55). Myeloprotection efficacy outcomes mirrored those in the pivotal study, with the trilaciclib group demonstrating statistically significant improvements in DSN in cycle 1 and the occurrence of SN compared with the placebo group. Additional supportive neutrophil-related endpoints all favored trilaciclib, and a similar trend for improved RBC endpoints was observed, including a statistically significant reduction in the percentage of patients receiving RBC transfusions (55). Fewer patients experienced cycle delays and dose reductions with trilaciclib versus placebo, and trilaciclib improved the safety profile of E/P, as evidenced by a reduction in the occurrence of high-grade AEs, including hematologic AEs (55).

A third trial was performed to explore the myeloprotective effects of trilaciclib in the setting of a more myelosuppressive chemotherapy, and to evaluate its effects when administered to patients with HSPCs that have already been damaged by chemotherapy (56). Patients with ES-SCLC received trilaciclib or placebo prior to second- or third-line topotecan. Administration of trilaciclib provided myeloprotection benefits across multiple lineages, with fewer high-grade hematologic toxicities, particularly neutropenia and anemia. Patients receiving trilaciclib completed more cycles and had fewer dose reductions compared with those receiving placebo. Again, antitumor efficacy was similar between treatment groups.

All three SCLC trials included patient-reported outcome measures as exploratory endpoints. Overall, patients receiving trilaciclib reported significant improvements in several areas of health-related QoL, including physical and functional well-being, symptoms and impact of fatigue, and symptoms and effects on physical and functional well-being due to anemia (58). This finding may be especially pertinent in light of COVID-19, which is likely to further exacerbate the impact of myelosuppression on health-related QoL by heightening fears that a weakened immune system will put patients at risk of severe complications from the virus.

Several other key findings from these trials are particularly relevant in the COVID-19 era. Reductions in DSN in cycle 1 and the occurrence of SN are notable given that CIN is associated with COVID-19 complications (36). Furthermore, a subanalysis of data pooled from all three studies showed an approximate six-fold decrease in the incidence of hospitalizations due to myelosuppression or sepsis in patients receiving trilaciclib versus placebo (58). Mitigation of myelosuppression with trilaciclib may therefore reduce the need for hospitalization or the length of hospital stay, thereby reducing the risk of nosocomial infections, including COVID-19. Patients receiving trilaciclib also required fewer RBC transfusions, which could be beneficial given the potential for reduced availability of blood products during the ongoing pandemic (33). Finally, the reduced need for growth factor and transfusion support may limit the need for outpatient visits to health care facilities and may reduce the risk of transfusion-related immunosuppression (19).

In addition to the SCLC trials, a randomized, open-label, phase 2 study of trilaciclib prior to gemcitabine and carboplatin (GCb) was performed in patients with metastatic triple-negative breast cancer, predominantly a functionally CDK4/6-independent disease (53). Patients with metastatic triple-negative breast cancer who had received ≤ 2 previous lines of chemotherapy in the recurrent/metastatic setting were randomized to receive GCb on days 1 and 8, trilaciclib prior to GCb on days 1 and 8, or trilaciclib alone on days 1 and 8 and prior to GCb on days 2 and 9, in 21-day cycles. Administering trilaciclib increased the duration of exposure and cumulative dose of chemotherapy; however, despite patients in the trilaciclib groups receiving more chemotherapy, rates of hematologic AEs were comparable between treatment arms. In this study, there was no statistically significant improvement in DSN or occurrence of SN; however, patients receiving trilaciclib prior to GCb had improved overall survival.

The manifestation of clinically significant benefit with trilaciclib as a myeloprotection therapy and/or enhancement of antitumor efficacy may depend on the clinical context in which trilaciclib is administered, i.e., the type or schedule of chemotherapy (and trilaciclib), tumor type, and host factors. The myeloprotective effects of trilaciclib are predicted to be influenced primarily by the host and the degree of myelosuppression associated with the chemotherapy regimen, such that the most significant effects on myelosuppression occur with sequential-day, cyclical chemotherapy regimens given to chemotherapy-naïve patients. By contrast, the effects of trilaciclib on antitumor efficacy are predicted to be an immune-mediated event influenced by the tumor type and host, along with the chemotherapy type and schedule, whereby the most significant effects on antitumor efficacy are predicted to occur with more immunogenic chemotherapy regimens, tumors that are sensitive to immune modulation, and a favorable host immune system.

The potential benefits of trilaciclib will be further investigated in two pivotal phase 3 trials in colorectal cancer (NCT04607668) and triple-negative breast cancer (NCT04799249), two phase 2 trials in non-small cell lung cancer (NSCLC; NCT04863248) and bladder cancer (NCT04887831), and as part of the I-SPY 2 neoadjuvant trial in patients with breast cancer (NCT01042379).

## Plinabulin

Plinabulin is an intravenous, marine-derived small molecule that promotes microtubule destabilization, leading to activation of guanine nucleotide exchange factor-H1. This triggers downstream molecular pathways that drive dendritic cell maturation and antigen-induced T-cell activation, thereby improving antitumor immunity (59, 60). In addition to its immune-potentiating effects (60, 61), plinabulin can prevent CIN caused by docetaxel by boosting the number of HSPCs in the bone marrow and has demonstrated evidence of alleviating CIT (62–70).

In the phase 2 part of a phase 2/3 study of plinabulin plus docetaxel versus pegfilgrastim plus docetaxel in patients with NSCLC, plinabulin showed a similar effect on CIN compared with pegfilgrastim, but only plinabulin reduced the incidence of thrombocytopenia; plinabulin was also associated with less bone pain (63). A significant QoL improvement was observed with plinabulin across three of four parameters measured (global health status, symptom scales, and summary score), with additional improvements in fatigue, pain, and insomnia symptom scales (71). Plinabulin is currently in phase 3 development for the prevention of docetaxel-induced neutropenia (versus pegfilgrastim) in patients with solid tumors (NCT03102606; Protective-1), for the prevention of docetaxel, doxorubicin and cyclophosphamide (TAC)-induced neutropenia (in combination with pegfilgrastim) in patients with breast cancer (NCT03294577; Protective-2), and for the treatment of CIN (versus pegfilgrastim) in patients with advanced NSCLC (NCT02504489; DUBLIN-3). Preliminary results from Protective-1 were similar to those from phase 2/3 trial in NSCLC, with single-agent plinabulin showing non-inferior protection against CIN compared with pegfilgrastim, but having advantages with respect to bone pain, thrombocytopenia, and convenience (same day vs next day dosing) over pegfilgrastim, as well as demonstrating anticancer activity (66). In September 2020, plinabulin received Breakthrough Therapy Designation for the prevention of CIN in both the US and China (67), based on positive interim analysis results from the phase 3 breast cancer trial. Topline data from Protective-2 have since been reported, showing that plinabulin in combination with pegfilgrastim significantly improved the rate of prevention of Grade 4 neutropenia in cycle 1 (primary endpoint) compared with pegfilgrastim alone, and achieved statistical significance in all key secondary endpoints, including DSN and absolute neutrophil count (ANC) nadir in cycle 1 (68, 69). Addition of plinabulin to pegfilgrastim also reduced the rate of profound neutropenia (ANC <0.1 cells x 10^9^/L) by more than 50%, as well as its clinical sequelae in the form of FN and hospitalizations (70).

Similar to findings with trilaciclib, both the reduced incidence of hospitalizations and reductions in the occurrences of severe/profound neutropenia with plinabulin are notable when considering the risks of healthcare-associated transmission of COVID-19, and COVID-19 complications associated with CIN (29, 36, 72). Considering the need to reduce hospitalizations and emergency room visits during the COVID-19 pandemic, an analysis of combined data from the Protective-1 and Protective-2 trials was conducted to evaluate the effects of plinabulin on CIN and its consequences. Compared with pegfilgrastim, the use of plinabulin required at least 50% fewer interactions with the health care system and was equally effective in preventing CIN and its clinical sequelae, while also resulting in less thrombocytopenia and bone pain (73). Thus, like trilaciclib, plinabulin may limit the need for visits to health care facilities in the COVID-19 era, as well as potentially helping to reduce the demand for blood product supplies by reducing the incidence of thrombocytopenia.

## Avatrombopag

Avatrombopag (DOPTELET^®^) is an orally administered thrombopoietin receptor agonist that stimulates proliferation and differentiation of megakaryocytes from HSPCs, resulting in increased platelet production (74, 75). Avatrombopag is approved in the US for the treatment of thrombocytopenia in adult patients with chronic liver disease who are scheduled to undergo a procedure, and for the treatment of thrombocytopenia in adults with chronic immune thrombocytopenia who have had an insufficient response to a previous treatment (75). Two phase 3 trials of avatrombopag for periprocedural thrombocytopenia in patients with chronic liver disease showed that avatrombopag reduced the need for rescue procedures such as platelet transfusion. Likewise, phase 2 and 3 trials in patients with immune thrombocytopenia showed that avatrombopag led to increased platelet count and longer duration without rescue therapy (74, 75). In December 2019, avatrombopag was granted Orphan Drug Designation for the potential treatment of CIT (76). However, a phase 3 trial (NCT03471078) investigating avatrombopag in patients with CIT receiving chemotherapy for ovarian, lung (SCLC and NSCLC), and bladder cancer did not meet the composite primary endpoint of avoiding platelet transfusions, chemotherapy dose reductions by ≥ 15%, and chemotherapy dose delays by ≥ 4 days (77).

## Romiplostim

Romiplostim (NPLATE^®^) is a subcutaneous Fc–peptide fusion protein that binds to and activates the thrombopoietin receptor, leading to increased platelet production (78, 79). It is approved and widely used for the treatment of immune thrombocytopenia (78, 79), and is under investigation for CIT in patients with various tumor types (79). In a phase 2 trial of romiplostim in patients with solid tumors with CIT, 93% of romiplostim-treated patients experienced correction of their platelet count within 3 weeks, compared with only 12.5% of control-treated patients; most patients treated with romiplostim were able to resume chemotherapy without recurrence of CIT (79). Additionally, a retrospective study of patients with solid tumors or non-myeloid hematologic malignancies who received off-label romiplostim found that 71% of patients with solid tumors achieved a romiplostim response (median on-romiplostim platelet count ≥75×10^9^/L and ≥30×10^9^/L higher than baseline), and 79% and 89% of patients with solid tumors avoided further chemotherapy dose reductions/delays and platelet transfusions, respectively (80). Several additional prospective trials are ongoing to evaluate romiplostim for the treatment of CIT in patients with non-hematologic cancers (phase 2; NCT02052882), gastrointestinal or colorectal cancer (phase 3; NCT03362177), and NSCLC, ovarian cancer, or breast cancer (phase 3; NCT03937154). Although there is little evidence regarding optimal management strategies for thrombocytopenia in the setting of COVID-19, two recent cases have been reported of patients with acute thrombocytopenia secondary to COVID-19 successfully treated with romiplostim (81, 82). A 67-year-old male patient developed severe thrombocytopenia complicated by subdural hematoma and rectal bleed, associated with COVID-19. Despite receiving platelet transfusions, his platelet count did not increase, and he was non-responsive to steroids and intravenous immunoglobulins. Eltrombopag (another thrombopoietin receptor agonist) was given but stopped as the patient had difficulty swallowing the tablets. Subsequent treatment with romiplostim resulted in gradual recovery to a normal platelet count (81). The second patient was a 6-year-old male with a complex medical history, including mild chronic thrombocytopenia. Following a further reduction in platelet count that was associated with SARS-CoV-2 infection, he received romiplostim to stimulate platelet production, which resulted in the resolution of spontaneous bleeding and improvement of platelet counts (82). Based on these (albeit limited) findings, it is feasible that the use of romiplostim may also be of benefit in preventing or treating acute thrombocytopenia in patients receiving myelosuppressive chemotherapy in a COVID-19/post-COVID-19 setting, including those with existing COVID-19 infection.

## ALRN-6924

ALRN-6924 is an intravenously administered, stabilized, cell-permeating peptide that disrupts interactions between the p53 tumor suppressor protein and its endogenous inhibitors, mouse double minute 2 and mouse double minute X (83). ALRN-6924 blocks wild-type p53 cells, including normal bone marrow cells, in the S phase, thereby potentially preventing chemotherapy-induced toxicity while preserving or enhancing efficacy against p53-mutant cancers. A preclinical study demonstrated that ALRN-6924 induces transient, reversible cell cycle arrest in bone marrow cells *in vitro* and *in vivo* and protects human bone marrow cells against topotecan-induced myelosuppression (83). The ability of ALRN-6924 to mitigate topotecan-induced anemia, neutropenia, and thrombocytopenia is being investigated in a phase 1/2 trial in patients with SCLC (NCT04022876), with plans to include an additional phase 1b cohort of patients with NSCLC treated with docetaxel, and a randomized expansion SCLC cohort using alternating chemotherapy with and without ALRN-6924. Preliminary results in patients with *p53*-mutated SCLC demonstrated that ALRN-6924 treatment 24 hours prior to administration of second-line topotecan resulted in lower rates of severe anemia, thrombocytopenia, and neutropenia compared with historical data (84). If ALRN-6924 proves to be effective for preventing topotecan-induced anemia, thrombocytopenia, and neutropenia, it could also be a useful alternative to conventional supportive care interventions for cytopenias caused by myelosuppressive chemotherapy in a post-COVID-19 setting.

## Roxadustat

Roxadustat is a first-in-class, oral inhibitor of hypoxia-inducible factor prolyl hydroxylase that promotes erythropoiesis by increasing endogenous production of erythropoietin (85). Roxadustat has been investigated for the treatment of anemia associated with chronic kidney disease in three global phase 3 trials and is approved in this indication in China and Japan (85, 86). Pooled analyses of phase 3 studies comparing roxadustat with placebo in patients with non–dialysis-dependent chronic kidney disease and with epoetin alfa in dialysis-dependent patients showed that roxadustat was at least as efficacious in correcting anemia and had a better cardiovascular safety profile compared with placebo and epoetin alfa (87). Notably, a separate phase 3 trial in patients undergoing dialysis in China found that, unlike epoetin alfa, which increased hemoglobin levels to a greater extent in patients with normal C-reactive protein (CRP) levels compared with those with elevated CRP levels, the hemoglobin response with roxadustat appeared to be unaffected by CRP levels (a marker of inflammation) (44). Thus, roxadustat may be more effective than ESAs in treating anemia in an inflammatory milieu such as that observed in COVID-19 (41). The efficacy and safety of roxadustat in the treatment of CIA in patients with non-myeloid malignancies is being evaluated in a single-arm phase 2 trial (NCT04076943).

## Conclusions

Myelosuppression is a common dose-limiting side effect of cytotoxic chemotherapy that leads to considerable morbidity and negatively impacts treatment outcomes and QoL. In the context of the COVID-19 pandemic, myelosuppression poses an additional threat by placing patients at an increased risk of exposure to the virus and infection, while also increasing the likelihood of severe infectious complications. Current supportive care measures for myelosuppression only apply to a single hematopoietic lineage, do not spare the bone marrow from cytotoxicity, and can often lead to AEs such as bone pain and thrombotic events. Fortunately, newer agents are available or in development that may help to overcome some of these limitations. By mitigating or preventing the toxic effects of chemotherapy and enabling broad HSPC protection, these agents may enable a paradigm shift in the management of patients treated with myelosuppressive chemotherapy regimens.

## Author Contributions

All named authors meet the International Committee of Medical Journal Editors criteria for authorship for this article. All authors contributed to the article and approved the submitted version.

## Conflict of Interest

GL reports grants from Amgen, and personal fees from BeyondSpring and G1 Therapeutics, Inc. He has consulted for Invitae, Spectrum, Samsung, and Partners. NK reports personal fees from BeyondSpring, Bristol-Myers Squibb, Celldex, Invitae, Janssen, Spectrum, and Total Health. MA reports personal fees and non-financial support from the Multinational Association for Supportive Care in Cancer, European Society of Medical Oncology, and the European CanCer Organisation, grants and personal fees from Helsinn and Sandoz, and personal fees from G1 Therapeutics, Kyowa Kirin, Merck USA, Pfizer, Taiho, Tesaro, and Vifor.
